# Paeoniflorin Alleviates Cisplatin-Induced Diminished Ovarian Reserve by Restoring the Function of Ovarian Granulosa Cells via Activating FSHR/cAMP/PKA/CREB Signaling Pathway

**DOI:** 10.3390/molecules28248123

**Published:** 2023-12-15

**Authors:** Qingchang Wu, Miao Chen, Yao Li, Xiangyun Zhao, Cailian Fan, Yi Dai

**Affiliations:** 1 Institute of Traditional Chinese Medicine & Natural Products, College of Pharmacy/Guangdong Province Key Laboratory of Pharmacodynamic Constituents of TCM and New Drugs Research/International Cooperative Laboratory of Traditional Chinese Medicine Modernization and Innovative Drug Development of Ministry of Education (MOE) of China, Jinan University, Guangzhou 510632, China; wuqingchangjnu@163.com (Q.W.); cm1998321@163.com (M.C.); llly0809@163.com (Y.L.); 2 College of Medicine, Henan Engineering Research Center of Funiu Mountain’s Medicinal Resources Utilization and Molecular Medicine, Pingdingshan University, Pingdingshan 467000, China; 15090335325@163.com

**Keywords:** paeoniflorin, diminished ovarian reserve, ovarian granulosa cell, FSHR/cAMP/PKA/CREB signaling pathway

## Abstract

Paeoniflorin (PAE) is the main active compound of Radix Paeoniae Rubra (a valuable traditional Chinese medicine and a dietary supplement) and exerts beneficial effects on female reproductive function. However, the actions of PAE on diminished ovarian reserve (DOR, a very common ovarian function disorder) are still unclear. Herein, our study investigated the effect and potential mechanism of PAE on DOR by using cisplatin-induced DOR mice and functional impairment of estradiol (E_2_) synthesis of ovarian granulosa-like KGN cells. Our data show that PAE improved the estrous cycle, ovarian index, and serum hormones levels, including E_2_, and the number of antral follicles and corpora lutea in DOR mice. Further mechanism results reveal that PAE promoted aromatase expression (the key rate-limiting enzyme for E_2_ synthesis) and upregulated the FSHR/cAMP/PKA/CREB signaling pathway in the ovaries. Subsequently, PAE improved the levels of E_2_ and aromatase and activated the FSHR/cAMP/PKA/CREB signaling pathway in KGN cells, while these improving actions were inhibited by the siRNA-FSHR and FSHR antagonist treatments. In sum, PAE restored the function of E_2_ synthesis in ovarian granulosa cells to improve DOR by activating the FSHR/cAMP/PKA/CREB signaling pathway, which exhibited a new clue for the development of effective therapeutic agents for the treatment of DOR.

## 1. Introduction

Radix Paeoniae Rubra (RPR), the dried roots of *Paeonia lactiflora* Pall or *Paeonia veitchii* Lynch of Family Ranunculaceae, has been listed as a dietary supplement by the National Health Commission of the People’s Republic of China and can exert effective action against gynecological disease, such as menstrual disorders [1,2,3], and also exhibits a beneficial effect in ovarian aging [4]. Paeoniflorin (PAE), a monoterpene glucoside of the total glycoside of paeony and the main active compound of RPR, has been verified as a safe extraction in clinical pharmacological studies [5]. In the literature, there are numerous reports that PAE presents many biological activities, including neuroprotective and anti-depressive actions [6], anti-inflammatory and immunoregulatory effects [7], modulating oxidative stress and hepatic fibrosis [8], etc. Importantly, recent studies reported that PAE exerted protective actions of rat ovary on ischemia reperfusion injury [9], improved ovulation in female rats with polycystic ovary syndrome [10], and enhanced endometrial receptivity in female mice [11]. Therefore, PAE has attracted more and more attention for its beneficial effects on female reproductive function.

Diminished ovarian reserve (DOR), a very common ovarian function disorder, refers to a reduction in the number of mature ovarian follicles and a decrease in oocyte quality, which causes a deficiency of sex hormones, such as estradiol (E_2_), and a decrease in female fertility [12,13]. The prevalence of DOR is approximately 26% [14], and the clinical symptoms of DOR patients mainly include oligomenorrhea, hypomenorrhea, amenorrhea, and infertility [15,16]. DOR severely affects the quality of life and can develop into premature ovarian failure without effective treatment [12]. At present, common DOR treatments, such as hormone replacement therapy (HRT), can maintain regular menstruation, but these methods are hardly satisfactory with diverse side effects, including breast cancer [17,18]. In addition, although assisted reproductive technology presents a vital role in reproductive medicine, DOR is still one of the hardest challenges for it [19]. Therefore, seeking more safe and effective therapeutic agents for the treatment of DOR is a meaningful task.

It is widely known that the development of DOR is closely related to folliculogenesis, which relies on the precise regulation of a variety of cells [20,21]. More and more studies have indicated that the dysfunction of ovarian granulosa cells was one of the most important factors for the pathogenesis of DOR [22,23]. Ovarian granulosa cells, a main functional cell in the ovaries, provide nutrients such as E_2_ to promote the development and maturation of oocytes [24,25]. More specifically, the follicle-stimulating hormone (FSH), secreted by basophils in the anterior pituitary, can combine with the follicle-stimulating hormone receptor (FSHR) in ovarian granulosa cells to activate the cascade amplification of downstream signal molecules, including cyclic adenosine monophosphate (cAMP), protein kinase A (PKA), and cAMP response element-binding protein (CREB), which further regulates the expression of aromatase to catalyze the transformation of androgens into E_2_ [26,27]. Importantly, the expression of the FSHR and cAMP signaling pathways has been proven to exhibit vital actions in regulating follicular development [28,29,30,31]. Therefore, regulating the FSHR/cAMP/PKA/CREB signaling pathway to improve the function of ovarian granulosa cells may be an effective approach for the treatment of DOR.

Undoubtedly, PAE exhibits a wide range of beneficial actions on female reproductive function, yet the action and the potential mechanism of PAE on DOR at present have not been defined. Herein, the biological activity and related signaling pathway of PAE on DOR were explored by using a DOR mouse model induced by cisplatin (CDDP) and using an in vitro model with CDDP-induced functional impairment of E_2_ synthesis in ovarian granulosa-like KGN cells. Meanwhile, the exact mechanism of PAE was further revealed in vitro by using small-interfering RNA (siRNA) and the antagonist.

## 2. Results

### 2.1. PAE Supplementation Improved Estrous Cycle and Ovarian Index in DOR Mice

**Figure 1:**
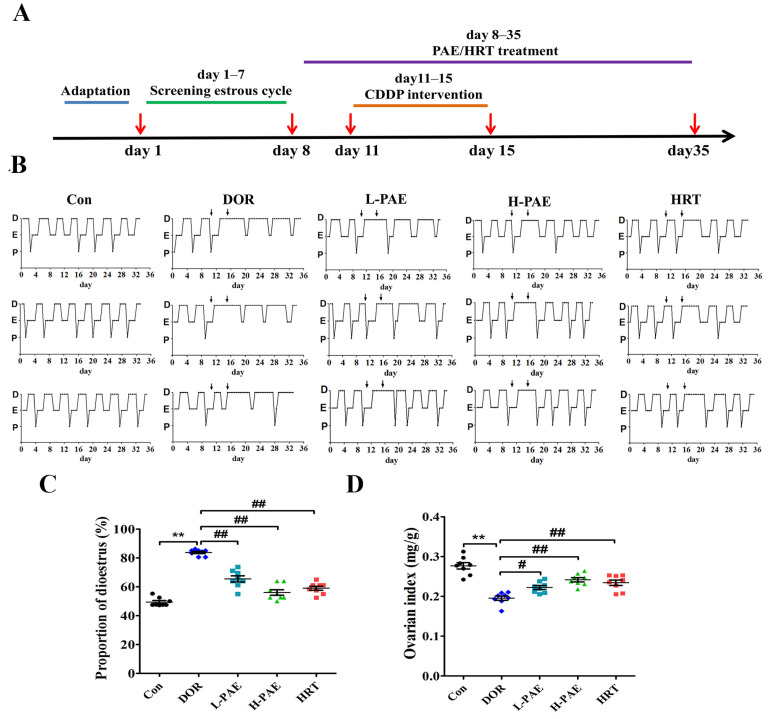
PAE supplementation improved estrous cycle and ovarian index in CDDP-induced DOR mice. (**A**) Experimental protocol of the action of PAE on DOR mice. (**B**) Representative estrous cycle in each group, and downward arrows represent the period of CDDP treatment for 5 days. (**C**) The proportion of dioestrus from day 8 in each group. (**D**) Ovarian indexes in each group. P: proestrus, E: estrus, and D: dioestrus. *n* = 8 per group. ** *p* < 0.01 vs. Con group; ^#^ *p* < 0.05, ^##^ *p* < 0.01 vs. DOR group.

Oligomenorrhea, hypomenorrhea, and amenorrhea are the most common symptoms of DOR patients [15,16]. Similar to the menstrual cycle in women, the regular estrous cycle is one of the main observations of normal ovarian function in female mice. The experimental protocol is shown in Figure 1A. From day 1 to day 7, vaginal smears were collected to observe the estrous cycle, and mice with normal estrous cycles were screened to randomly assign into five groups: Control (Con), Model (DOR), Low-dose paeoniflorin (L-PAE), High-dose paeoniflorin (H-PAE), and Hormone replacement therapy (HRT). According to the data of our previous preliminary experiments, from day 8, all mice were administered intragastrically with paeoniflorin or distilled water for 4 weeks. Meanwhile, the mice in the DOR, L-PAE, H-PAE, and HRT groups received CDDP (1.0 mg/kg/day) intraperitoneal injection from day 11 to day 15, and the effects of CDDP or PAE on estrous cycle were investigated by vaginal smears. The data of the estrous cycle in Figure 1B reveal that dioestrus (D) in the Con group of mice generally lasted 2–3 days, while in the DOR group of mice, it was obviously prolonged after CDDP treatment, which indicated that CDDP treatment strongly affected ovarian function in DOR mice. Meanwhile, the prolonged period of dioestrus (D) was gradually returned to 2–3 days in the H-PAE group, and HRT treatment also presented positive actions that restored a regular estrous cycle. Furthermore, the proportion of dioestrus (D) from the first day of CDDP treatment (day 11) to the day of euthanasia (day 35) was calculated according to the data of Figure 1B. DOR mice exhibited an increasing proportion of dioestrus (D), while PAE treatment, especially in the H-PAE group, largely restored the proportion to the level of normal estrous cycles (Figure 1C, *p* < 0.01). In addition, the ovarian index is the most direct index that reflects the effects of drugs on the ovaries. As exhibited in Figure 1D, compared with the Con group, CDDP largely decreased the ovarian index by 29.3% in DOR mice (*p* < 0.05). The L-PAE, H-PAE, and HRT groups significantly increased the ovarian index by 13.7%, 23.6%, and 19.8% compared to DOR mice (*p* < 0.05). These data suggest that PAE supplementation exerted beneficial effects to restore ovarian function.

### 2.2. PAE Supplementation Upregulated the Number of Antral Follicles and Corpora Lutea in DOR Mice to Varying Degrees

**Figure 2:**
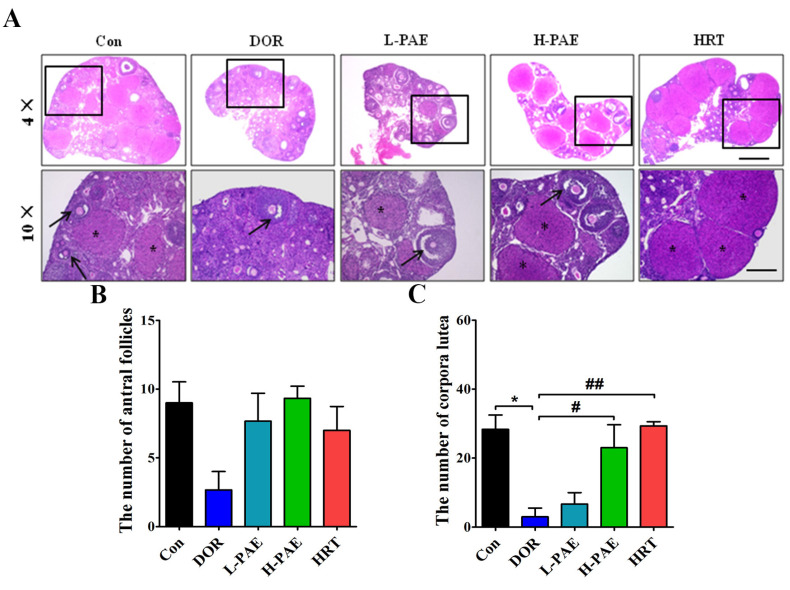
PAE supplementation upregulated the number of antral follicles and corpora lutea in DOR mice to varying degrees. (**A**) Hematoxylin and eosin (H&E) staining of the ovaries in each group with 4× and 10× magnification; asterisks represent corpora lutea, and arrows represent antral follicles, and the scale bar is 300 μm. (**B**) The number of antral follicles in each group. (**C**) The number of corpora lutea in each group. *n* = 3 per group. * *p* < 0.05 vs. Con group; ^#^ *p* < 0.05, ^##^ *p* < 0.01 vs. DOR group.

DOR patients exhibit a decreasing number of mature ovarian follicles, and antral follicle and corpus luteum are essential processes of follicular maturation [12,13]. Our data show that CDDP treatment to some extent caused a decreasing number of antral follicles (Figure 2A,B) and markedly reduced the number of corpora lutea by 89.4% compared with the Con group (Figure 2A–C, *p* < 0.05), indicating that CDDP exhibited destructive effects on follicular development. Meanwhile, the number of antral follicles was partially increased after L-PAE and H-PAE treatment, and the number of corpora lutea was largely increased in the H-PAE group and HRT group (Figure 2A–C, *p* < 0.05), respectively. In sum, these results suggest that PAE treatment presented positive actions to restore the disorder of follicular development and ovulation in DOR mice.

### 2.3. PAE Supplementation Restored the Balance of Serum Hormones in DOR Mice

**Figure 3:**
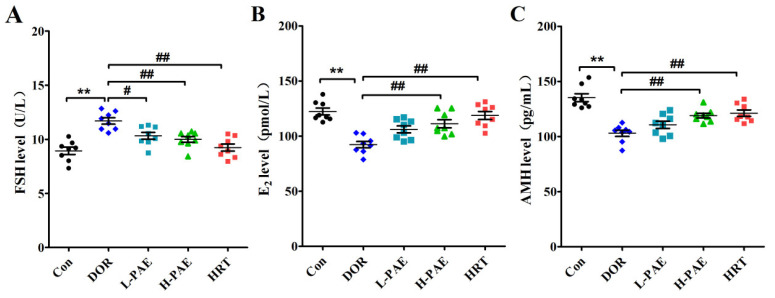
PAE supplementation restored the balance of serum hormones in DOR mice. (**A**) The levels of serum FSH in each group. (**B**) The levels of serum E_2_ in each group. (**C**) The levels of serum AMH in each group. *n* = 8 per group. ** *p* < 0.01 vs. Con group; ^#^ *p* < 0.05, ^##^ *p* < 0.01 vs. DOR group.

DOR patients commonly present abnormal serum hormone levels, including a high serum FSH level and low serum E_2_ and anti-Müllerian hormone (AMH) levels [12,13]. To further evaluate the effects of PAE on serum hormones, the levels of serum FSH, E_2_, and AMH in mice were measured by using an enzyme-linked immunosorbent assay (ELISA) kit assay. The results reveal that the level of serum FSH in the DOR group was markedly increased by 30.8% (Figure 3A, *p* < 0.01), while the levels of serum E_2_ and AMH were largely decreased by 24.5% and 23.9% compared with Con group (Figure 3B,C, *p* < 0.01), respectively, suggesting that CDDP treatment could cause abnormal levels of serum hormones in mice. However, these adverse effects in serum hormones induced by CDDP were significantly improved after H-PAE intervention (Figure 3A–C, *p* < 0.01). These above results suggest that PAE positively regulated the balance of serum hormones to preserve the ovarian reserve function in DOR mice.

### 2.4. PAE Supplementation Upregulated FSHR/cAMP/PKA/CREB Signaling Pathway in the Ovaries of DOR Mice

**Figure 4:**
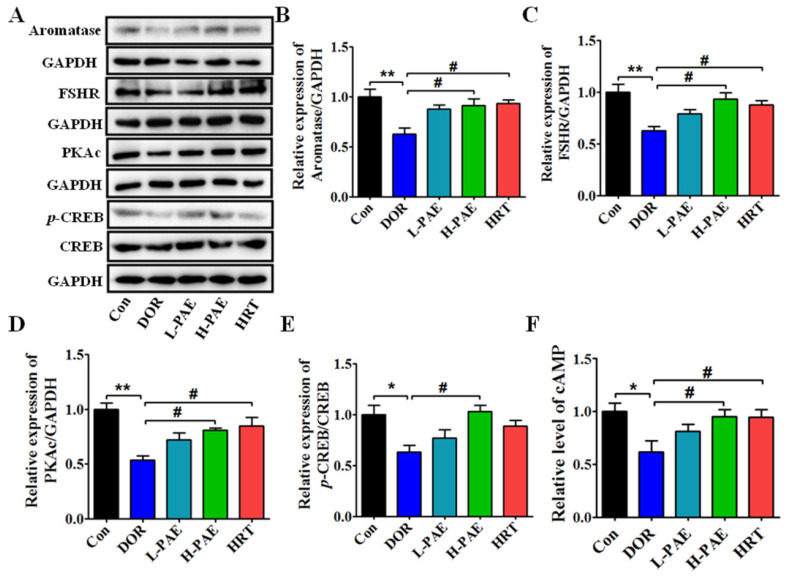
PAE upregulated FSHR/cAMP/PKA/CREB signaling pathway in the ovaries of DOR mice. (**A**) The expressions of aromatase, FSHR, PKAc, and *p*-CREB/CREB in the ovaries were detected by using Western blot. (**B**–**E**) Each immunoreaction band of aromatase, FSHR, PKAc, and *p*-CREB/CREB was normalized against GAPDH. (**F**) Relative level of cAMP in the ovaries. *n* = 3 per group. * *p* < 0.05, ** *p* < 0.01 vs. Con group; ^#^ *p* < 0.05 vs. DOR group.

Generally, the FSH binds to the FSHR in ovarian granulosa cells to activate the cascade amplification of downstream signal molecules of cAMP/PKA/CREB, which further actives the transcription of aromatase to catalyze the transformation of androgens into E_2_ [26,27]. Our previous results show that PAE intervention markedly improved the CDDP-induced decreasing levels of serum E_2_ in DOR mice. Next, our study investigated the actions of PAE on the expressions of aromatase (the key rate-limiting enzyme for the synthesis of E_2_) and the related signaling pathway. As displayed in Figure 4A,B, compared with the Con group, the expression of aromatase in the ovaries was reduced by 37.3% after CDDP treatment, while H-PAE supplementation could upregulate the expression of aromatase by 45.5% (*p* < 0.05), which was almost consistent with the results of serum E_2_. Furthermore, as shown in Figure 4C–F, the levels of FSHR, cAMP, protein kinase A catalytic subunit (PKAc), and phosphorylated cAMP response element-binding protein/cAMP response element-binding protein (*p*-CREB/CREB) in the ovaries of DOR mice were obviously decreased by 37.4%, 38.5%, 46.4%, and 36.6% compared with the Con group (*p* < 0.05), respectively, while the H-PAE group largely improved these effects by 49.5%, 54.6%, 50.7%, and 62.6% compared with the DOR group (*p* < 0.05), respectively, indicating that the FSHR/cAMP/PKA/CREB signaling pathway might play a key role in the actions of PAE.

### 2.5. PAE Restored the Function of E_2_ Synthesis in Ovarian Granulosa Cells by Activating FSHR/cAMP/PKA/CREB Signaling Pathway

**Figure 5:**
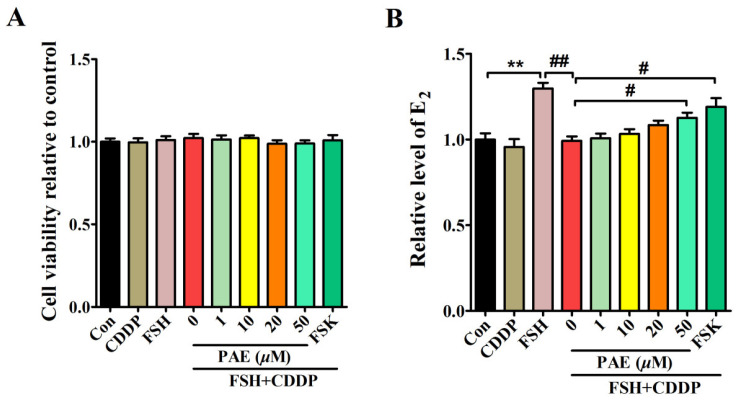
PAE treatment improved E_2_ synthesis in ovarian granulosa-like KGN cells. (**A**) Toxicity evaluation of PAE in KGN cells. (**B**) Effects of PAE on the levels of E_2_ in KGN cells. All data were obtained from at least three independent experiments. ** *p* < 0.01 vs. Con group; ^#^ *p* < 0.05, ^##^ *p* < 0.01 vs. 0 group.

**Figure 6:**
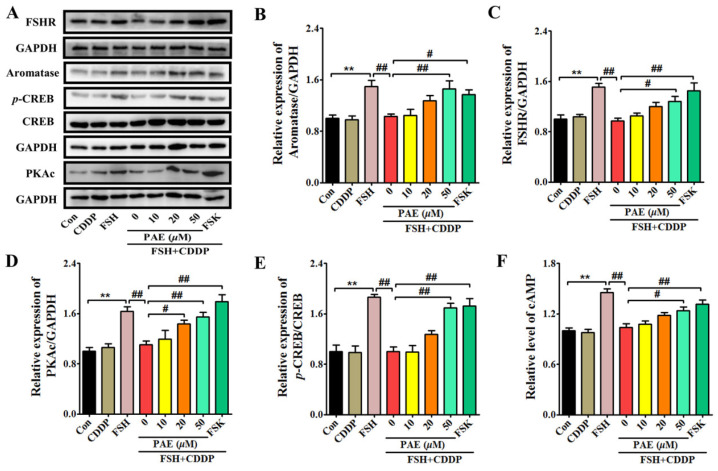
PAE treatment upregulated FSHR/cAMP/PKA/CREB signaling pathway in ovarian granulosa-like KGN cells. (**A**) The expressions of aromatase, FSHR, PKAc, and *p*-CREB/CREB in KGN cells were detected by using Western blot. (**B**–**E**) Each immunoreaction band of aromatase, FSHR, PKAc, and *p*-CREB/CREB was normalized against GAPDH. (**F**) Relative level of cAMP in KGN cells. All data were obtained from at least three independent experiments. ** *p* < 0.01 vs. Con group; ^#^ *p* < 0.05, ^##^ *p* < 0.01 vs. 0 group.

E_2_ synthesis is one of the most important functions of ovarian granulosa cells [24,25]. As exhibited in the in vivo results, DOR mice exhibited high levels of serum FSH, but decreasing levels of serum E_2_, indicating CDDP caused the dysfunction of ovarian granulosa cells in E_2_ synthesis, which was closely related to the inhibition of the FSHR/cAMP/PKA/CREB signaling pathway. To further elaborate the potential mechanism of PAE, an in vitro model was used that showed CDDP induced functional impairment of E_2_ synthesis in ovarian granulosa-like KGN cells. This model was established by cotreatment with FSH and CDDP according to the animal results and the reported literature [32]. As shown in Figure 5 and Figure 6A,B, the levels of E_2_ and protein expression of aromatase were obviously increased by 10 ng/mL FSH in KGN cells, while these effects were significantly inhibited after 1 µM CDDP cotreatment, indicating that the model of functional impairment of E_2_ synthesis in KGN cells was successfully established. Meanwhile, the levels of E_2_ and the expression of aromatase were dose-dependently increased by using PAE treatment for 48 h without obvious cytotoxicity, which suggested that PAE relieved CDDP-induced functional impairment of E_2_ synthesis in KGN cells, which was consistent with the in vivo results.

**Figure 7:**
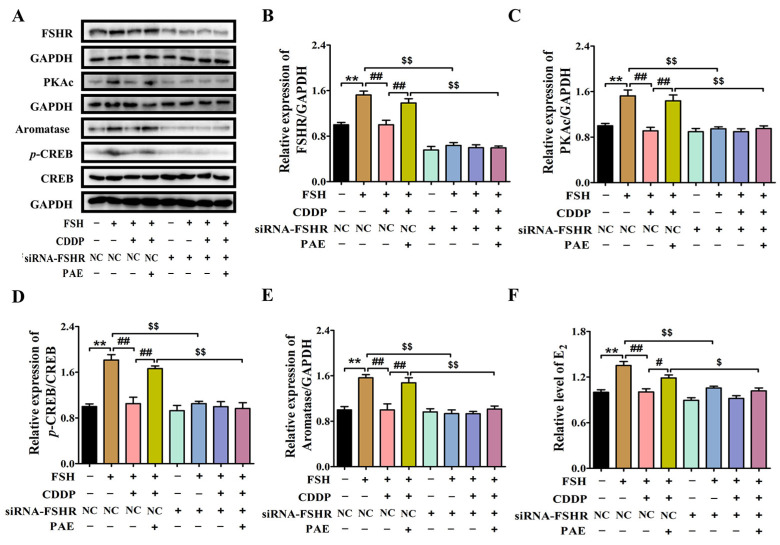
siRNA-FSHR transfection reduced the PAE-induced improving effects in ovarian granulosa-like KGN cells. (**A**) The expressions of FSHR, PKAc, *p*-CREB/CREB, and aromatase in KGN cells were detected by using Western blot. (**B**–**E**) Each immunoreaction band of FSHR, PKAc, *p*-CREB/CREB, and aromatase was normalized against GAPDH. (**F**) Effects of siRNA-FSHR transfection on the PAE-induced levels of E_2_. PAE: 50 µM. All results were obtained from at least three independent experiments. ** *p* < 0.01 vs. NC group; ^#^ *p* < 0.05, ^##^ *p* < 0.01 vs. NC + FSH + CDDP; ^$^ *p* < 0.05, ^$$^ *p* < 0.01 vs. the same tested group without siRNA-FSHR treatment.

**Figure 8:**
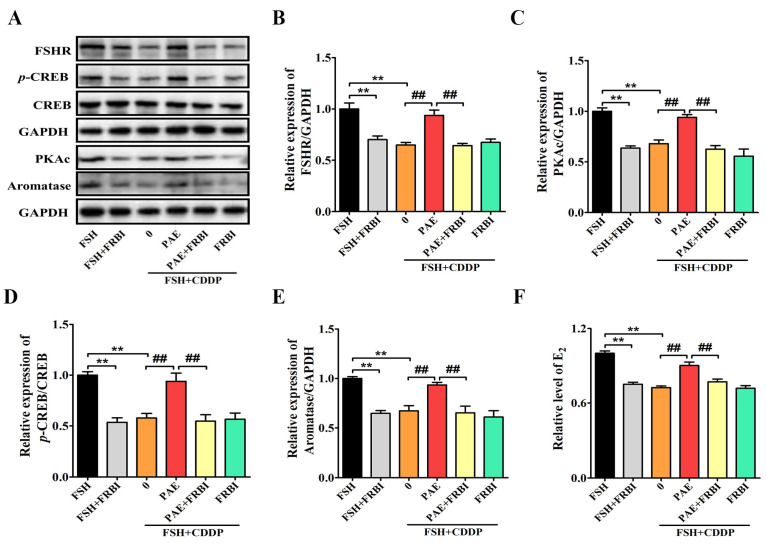
FSH receptor-binding inhibitor (FRBI) fragment (bi-10) inhibited the PAE-induced improving effects in ovarian granulosa-like KGN cells. (**A**) The expressions of FSHR, PKAc, *p*-CREB/CREB, and aromatase in KGN cells were detected by using Western blot. (**B**–**E**) Each immunoreaction band of FSHR, PKAc, *p*-CREB/CREB, and aromatase was normalized against GAPDH. (**F**) Effects of FRBI on the PAE-induced levels of E_2_. FRBI: 20 ng/mL. All results were obtained from at least three independent experiments. ** *p* < 0.01 vs. FSH group; ^#^ *p* < 0.05, ^##^ *p* < 0.01 vs. PAE group.

Interestingly, the FSH-induced levels of FSHR, cAMP, PKAc, and *p*-CREB/CREB were significantly decreased by 0.35-, 0.28-, 0.32-, and 0.46-fold after cotreatment with CDDP in KGN cells (Figure 6C–F, *p* < 0.05), respectively. Similar to the data of animal results, the levels of FSHR, cAMP, PKAc, and *p*-CREB/CREB were markedly increased by 0.31-, 0.19-, 0.40-, and 0.69-fold after PAE treatment (*p* < 0.05), respectively, and exhibited the same effects as forskolin (FSK, an activator of the cAMP signaling pathway) in activating the cAMP signaling pathway (Figure 6C–F). Subsequently, the study further investigated the roles of FSHR in the effects of PAE. As revealed in Figure 7A,B, the expression of FSHR was upregulated by PAE, while it was largely reduced by 0.57-fold after siRNA-FSHR transfection (*p* < 0.01). Meanwhile, the PAE-induced levels of PKAc, *p*-CREB/CREB, aromatase, and E_2_ were also obviously diminished by 0.33-, 0.41-, 0.31-, and 0.14-fold after siRNA-FSHR transfection (Figure 7C–F, *p* < 0.05), respectively. Importantly, as exhibited in Figure 8A–F, the PAE-induced upregulation of the FSHR/cAMP/PKA/CREB signaling pathway was also significantly decreased by the FSHR antagonist, FSH receptor-binding inhibitor (FRBI) fragment (bi-10). Collectively, these above data strongly indicate that PAE restored the function of ovarian granulosa cells in in vivo and in vitro by activating the FSHR/cAMP/PKA/CREB signaling pathway to improve CDDP-induced DOR in mice.

## 3. Discussion

Diminished ovarian reserve (DOR) is a disease of the female reproductive system, which presents a reduction in the number of mature ovarian follicles and a decrease in oocyte quality, high serum FSH level, and low serum E_2_ and AMH levels [12,13]. The pathogenesis of DOR is complex, and no unified animal model could be used on the study of DOR. Chemotherapy for oncological reasons is one of the common causes of DOR [19]; thus, cisplatin (CDDP, a chemotherapeutic drug) has been reported to be one of the most common methods to induce DOR with a shorter modeling cycle and typical pathological changes [33,34]. In the study, 1.0 mg/kg/day CDDP treatment for 5 consecutive days could cause an estrous cycle disorder, a decrease in the number of antral follicles and corpora lutea, an increase in the serum FSH level, and a decrease in the serum E_2_ and AMH levels in mice, which were largely consistent with the clinical features of DOR patients, indicating that the in vivo model was successfully established and suitable to be used on the study of DOR. Meanwhile, paeoniflorin (PAE) is the main active compound of Radix Paeoniae Rubra (a valuable traditional Chinese medicine and a dietary supplement) and it could, to varying degrees, restore the estrous cycle and the levels of serum hormones and increase the number of antral follicles and corpora lutea in DOR mice, which indicated that PAE improved the ovarian reserve in DOR mice and further supported the positive actions of PAE in treating female reproductive diseases [9,10,11]. In addition, PAE intervention hardly exhibited any negative effects on the renal index in DOR mice (Figure S1). Collectively, these above data suggest that PAE is promising as an effective and safe drug for treating DOR.

Ovarian granulosa cells, key functional cells in the ovaries, provide nutrients such as E_2_ to maintain the development and maturation of oocytes [24,25]. FSH binding to FSHR activates cascade amplification of the downstream signaling molecules, such as cAMP in ovarian granulosa cells, which further stimulates PKA to active the phosphorylation of CREB and increases the level of E_2_ by upregulating the transcription of aromatase [35,36]. In our study, the model mice exhibited similar pathology features of DOR patients with abnormal FSH and E_2_ levels; meanwhile, aromatase (CYP19A1) expression was significantly decreased in the ovarian granulosa cells of DOR patients compared to women with a normal ovarian reserve [37], which was also observed in the comparison of the Con group of mice and the DOR group of mice in our animal experiment, suggesting the dysfunction of E_2_ synthesis in the ovarian granulosa cells of DOR patients and DOR mice. Therefore, to further elucidate the actions of PAE on DOR, an in vitro model for the functional impairment of E_2_ synthesis in ovarian granulosa-like KGN cells was constructed by cotreatment with FSH and CDDP based on these phenomena of animal experiments. Importantly, PAE improved the levels of E_2_ and the expression of aromatase in KGN cells, which were consistent with the in vivo data. In brief, our present work is the first in vivo study and in vitro study to reveal the effects of PAE on DOR.

Studies have reported that haploinsufficiency of the FSHR gene (FSHR−/−) could impair follicular formation and development to cause infertility in female mice, and FSHR+/− female mice exhibited increasing levels of serum FSH and LH, and the decreasing levels of serum E_2_, caused premature exhaustion of the gonadal reserve and progressive decline in reproductive capacity [28,29]. Meanwhile, the cAMP signaling pathway also exhibited vital protective actions in the ovaries [30,31]. Therefore, the FSHR/cAMP/PKA/CREB signaling pathway is critical for folliculogenesis and ovarian function. In our study, CDDP could inhibit the levels of E_2_, FSHR, cAMP, PKAc, *p*-CREB/CREB, and aromatase in the ovaries of DOR mice and KGN cells, indicating that the FSHR/cAMP/PKA/CREB signaling pathway was involved in the CDDP-induced functional impairment of E_2_ synthesis in ovarian granulosa cells. Meanwhile, PAE supplementation obviously reversed these damaging effects of CDDP and increased the levels of E_2_, while siRNA-FSHR transfection and FSHR antagonist largely reduced the PAE-induced improving actions of the FSHR/cAMP/PKA/CREB signaling pathway and E_2_ synthesis in KGN cells. These above results suggest that PAE could restore the function of E_2_ synthesis in ovarian granulosa cells to improve the ovarian reserve in DOR mice by activating the FSHR/cAMP/PKA/CREB signaling pathway.

In this study, FSHR siRNA and FSHR antagonist were used to explore the mechanisms of PAE at the cellular level; the extent of the role of PAE still needs to be fully clarified by performing more experiments with transgenic mice and by using proteomics and transcriptomic analysis. Additionally, more experiments in our future works, including primary culture and ovary culture, induced pluripotent stem cells (IPSCs), and differentiated ovarian granulosa cells, could be conducted to further elucidate the potency of PAE for the treatment of DOR.

## 4. Materials and Methods

### 4.1. Materials

Paeoniflorin (PAE, Cas Number 23180-57-6) was purchased from Shanghai Winherb Medical Science Co., Ltd. (Shanghai, China), and the purity was more than 98% as determined by using HPLC analysis. Cisplatin injection was purchased from Jiangsu Haosen Co., Ltd. (Lianyungang, China).

### 4.2. Animals and Treatment

SPF female Kunming mice (6 weeks old, 25–30 g) were obtained from Beijing Huafukang Biotechnology Co., Ltd. (Beijing, China). All mice were housed in SPF-grade environmental conditions with constant temperature (25 °C). Experimental protocol is shown in Figure 1A. From day 1 to day 7, 40 mice with normal estrous cycles were screened to randomly assign into five groups (*n* = 8): Control (Con), Model (DOR), Low-dose paeoniflorin (L-PAE), High-dose paeoniflorin (H-PAE), and Hormone replacement therapy (HRT). HRT group was treated with estradiol valerate tablets and medroxyprogesterone acetate tablets [38]. According to the data of our previous preliminary experiments, from day 8, L-PAE and H-PAE groups were administered intragastrically with 75 and 150 mg/kg/day paeoniflorin [10], respectively, while Con group and DOR group were administered with an equal volume of double-distilled water by gavage, continuously for 4 weeks. All mice in the DOR, L-PAE, H-PAE, and HRT groups received CDDP (1.0 mg/kg/day) intraperitoneal injections from day 11 to day 15, while Con group was treated with an equal volume of saline.

### 4.3. Determinations of Estrous Cycle, Body Weight, and Ovarian Index

Day 1 to day 35, vaginal smears were collected to observe the estrous cycle at 8 a.m. and 8 p.m., and cell types were further classified by using the microscope. Proestrus (P), estrus (E), and dioestrus (D) were determined by the predominance of lymphocytes, nucleated epithelial cells, or keratinocytes, respectively [39]. Before CDDP intervention, the body weight of mice was recorded to calculate the volume of CDDP, and mice were weighed every 3 days after CDDP treatment. The ovaries were collected and weighed to calculate the ovarian index after euthanasia: ovarian index = ovarian weight (mg)/body weight (g).

### 4.4. Histological Analysis and Follicle Classification

The ovaries in each group were treated with 4% paraformaldehyde and further embedded with paraffin. Five μm thick sections of ovaries were cut and stained with hematoxylin and eosin (H&E). Antral follicle presented an antrum, a theca layer, a multilayer of granulosa cells, and the oocyte, while corpus luteum was the vital stage after ovulation [40].

### 4.5. Serum Hormones and Ovarian cAMP Assay in Mice

To observe the ovarian reserve of mice in depth, the levels of serum estradiol (E_2_) were tested via using mouse-specific competitive-based enzyme-linked immunosorbent assay (ELISA) kits (EK-Bioscience, Shanghai, China), and the levels of serum follicle-stimulating hormone (FSH) and anti-Müllerian hormone (AMH) were tested by using mouse-specific ELISA kits (Mei-mian, Wuhan, China). In addition, the ovaries were ground with normal saline (1 mg/10 µL) and then centrifuged (3000 rpm, 10 min) to collect the supernatant, and the levels of cAMP in the ovaries of mice were detected via using mouse-specific cAMP ELISA kits (EK-Bioscience, China).

### 4.6. Cell Viability Assay

Human ovarian granulosa-like KGN cells exhibit a pattern similar to steroidogenesis in ovarian granulosa cells, thus allowing for the study of steroidogenesis in ovarian granulosa cells, including E_2_ synthesis [41]. In our study, KGN cells were obtained from RCB (RIKEN Cell Bank) and were cultured in phenol red-free Dulbecco’s modified Eagle medium/Ham’s F-12 nutrient mix medium (Invitrogen, Carlsbad, CA, USA) supplemented with 10% Lonsera fetal bovine serum (FBS, Montevideo, Uruguay) and 1% penicillin and steptomycin (Invitrogen, USA) at 37 °C in a humidified atmosphere of 95% air and 5% CO_2_. KGN cells (1 × 10^4^ cells/well) were plated on a 96-well plate, and the cell confluence reached about 70%, and the cells were treated with related drugs such as FSH (R&D, Park Rapids, MI, USA). The cell survival rate was measured via using methyl thiazolyl tetrazolium (MTT) assay. Absorbance was determined at 570 nm via using microplate reader (Bio-Tek, Winooski, VT, USA).

### 4.7. Measurement of E_2_ and cAMP in KGN Cells

KGN cells (2 × 10^5^ cells/well) were seeded onto 6-well plates and were cultured with different drugs according to different research aims. The cell culture medium was obtained and then centrifuged (1000 rpm, 5 min) to collect the supernatant, and the levels of E_2_ from each group were tested via using human-specific ELISA kits (EK-Bioscience, China). Meanwhile, KGN cells (1 × 10^7^ cells/mL) were lysed by repeated freeze–thaw cycles 3 times and then centrifuged (13,000 rpm, 15 min) to collect the intracellular fluid. The concentration of intracellular cAMP was detected via using human-specific cAMP ELISA kits (EK-Bioscience, China).

### 4.8. Knockdown of FSHR by siRNA Transfection

As shown in Figure S2, siRNA-FSHR (siRNA-1 Forward: GAGCUGAAUCUAAGCGAUATT; Reverse: UAUCGCUUAGAUUCAGCUCTT) was synthesized by Beijing Qingke Biotech Biological Co., Ltd. (Beijing, China) and significantly reduced FSHR expression by more than 50%. KGN cells (2 × 10^5^ cells/well) were plated on 6-well plates for 24 h and then were treated with siRNA-FSHR and the corresponding negative control siRNA with Lipofectamine™ 2000 (Thermo Fisher, Waltham, MA, USA) in serum-free medium for 6 h.

### 4.9. Western Blot Analysis

The proteins of the collected ovaries and KGN cells were lysed in RIPA lysis buffer (Beyotime, Shanghai, China), which contained protease inhibitor (Beyotime, China) and protein phosphatase inhibitor (Beyotime, China), and were collected after centrifuging at 12,000 rpm for 15 min. The concentration of protein was detected by using BCA assay kit (Beyotime, China). Equally amounts of protein extracts were separated by electrophoresis on 8–12% SDS-PAGE and then transferred to PVDF membranes (Millipore, Billerica, MA, USA). Nextly, the membranes were incubated overnight at 4 °C with the following primary antibodies: Anti-FSHR (1:1000, Beyotime, China), Anti-Aromatase (1:500, Beyotime, China), Anti-PKAc (1:1000, Servicebio, Wuhan, China), Anti-CREB (1:1000, Beyotime, China), Anti-phospho-CREB (1:1000, Beyotime, China), and Anti-GAPDH (1:2000, Servicebio, China). Finally, these membranes were incubated at room temperature with the goat-anti-rabbit IgG-HRP (Servicebio, China) secondary antibody for 2 h and visualized by using the Enhanced Chemiluminescence Kit (Bio-Rad, Winooski, VT, USA).

### 4.10. Statistical Analysis

All experimental data were derived from at least three animal samples or three independent experiments and denoted as mean ± S.E.M. Meanwhile, differences between two or more groups were analyzed statistically with one-way analysis of variance (ANOVA) using GraphPad Prism version 7.0. *p* < 0.05 was considered statistically significant.

## 5. Conclusions

**Figure 9:**
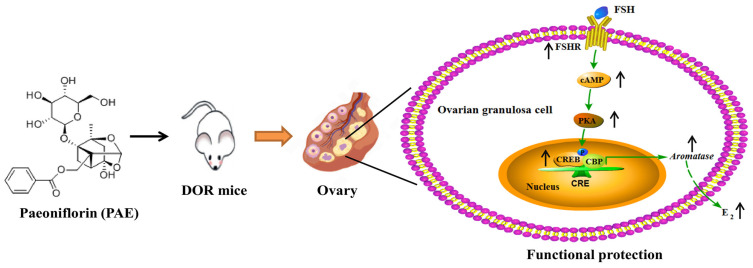
The potential mechanism involved in the effects of PAE in CDDP-induced DOR mice.

In conclusion, our study is the first research to report a novel effect of PAE against CDDP-induced DOR via activating the FSHR/cAMP/PKA/CREB signaling pathway in ovarian granulosa cells (Figure 9) and exhibits a new clue for the development of effective therapeutic agents for the treatment of DOR.

## Data Availability

The data presented in this study are available on request from the corresponding author.

## Supplementary Materials

The following supporting information can be downloaded at https://www.mdpi.com/article/10.3390/molecules28248123/s1. Figure S1: PAE intervention hardly exhibited negative effects on renal index in DOR mice (*n* = 8); Figure S2: Effect of FSHR siRNA transfection on gene expression of FSHR in KGN cells. * *p* < 0.05, ** *p* < 0.01 vs. NC-siRNA group.
