# Supplementary material for: Paeoniflorin Alleviates Cisplatin-Induced Diminished Ovarian Reserve by Restoring the Function of Ovarian Granulosa Cells via Activating FSHR/cAMP/PKA/CREB Signaling Pathway
Source: Molecules. 2023 Dec 15;28(24):8123. doi: 10.3390/molecules28248123 (PMC10745843; doi:10.3390/molecules28248123)
Supplement: Supplementary file 1 [file molecules-28-08123-s001.zip › molecules-2661740-supplementary.pdf]

Supplement Materials

40 mice with normal estrous cycle were screened to randomly assign into five groups (n = 8): Control (Con), Model (DOR), Low-dose paeoniflorin (L-PAE), High-dose paeoniflorin (H-PAE) and hormone replacement therapy (HRT). Renal index = renal weight (mg)/body weight (g).

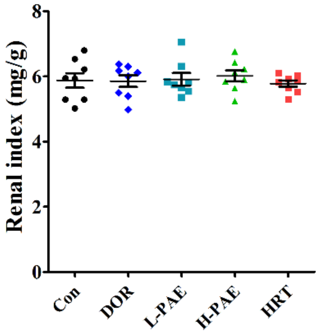

Figure S1. PAE intervention hardly exhibited negative effects on renal index in DOR mice (n = 8).

FSHR siRNA or control siRNA was transfected into KGN cells. siRNA-1 (Forward: GAGCUGAAUCUAAGCGAUATT; Reverse: UAUCGCUUAGAUUCAGCUCTT), siRNA-2 (Forward: CAGUUGAUAUCCAUACCAATT; Reverse: UUGGUAUGGAUAUCAACUGTT), siRNA-3 (Forward: GGCAAAUCUCUGAGCUUCATT; Reverse: UGAAGCUCAGAGAUU UGCCTT) were synthesized by Beijing Qingke Biotech Biological Co., Ltd (Beijing, China). KGN cells (2×10^5 cells/well) were plated at 6-well plates for 24 h, and then were treated with siRNA-FSHR and the corresponding negative control siRNA with Lipofectamine™ 2000 (Thermo Fisher) in serum-free medium for 6 h.

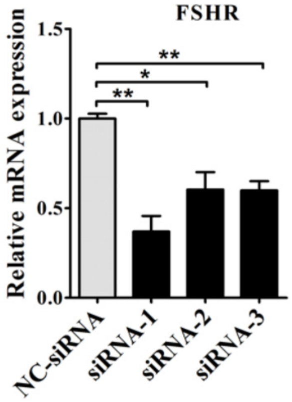

Figure S2. Effect of FSHR siRNA transfection on gene expression of FSHR in KGN cells. * $p < 0.05$, ** $p < 0.01$ vs. NC-siRNA group.
